# Correction: Stem cell factor restrains endoplasmic reticulum stress-associated apoptosis through c-Kit receptor activation of JAK2/STAT3 axis in hippocampal neuronal cells

**DOI:** 10.1371/journal.pone.0324301

**Published:** 2025-05-12

**Authors:** Haiying Shen, Junjie Nie, Guangqing Li, Hongyan Tian, Jun Zhang, Xiaofeng Luo, Da Xu, Jie Sun, Dongfang Zhang, Hong Zhang, Guifang Zhao, Weiyao Wang, Zhonghua Zheng, Shuyan Yang, Yuji Jin

The Funding statement for this article is incorrect. The correct Funding statement is as follows: This work was supported by “13th Five-Year” Science and Technology Project of Education Department of Jilin Province (JJKH20200456KJ); and Natural Science Foundation of Science and Technology Department of Jilin Province (20210101421JC).
