# Supplementary material for: Stem cell factor restrains endoplasmic reticulum stress-associated apoptosis through c-Kit receptor activation of JAK2/STAT3 axis in hippocampal neuronal cells
Source: PLoS One. 2024 Nov 15;19(11):e0310872. doi: 10.1371/journal.pone.0310872 (PMC11567559; doi:10.1371/journal.pone.0310872)
Supplement: Attachment — Submitted filename: review plos one.docx [file pone.0310872.s006.docx]

The study demonstrates that SCF inhibits L-Glu-induced ER stress-associated apoptosis in HT22 hippocampal neuronal cells and primary hippocampal neurons from APP/PS1 mice via the JAK2/STAT3 axis, suggesting potential therapeutic targets for both sporadic and familial Alzheimer's disease.

The study is well designed and has consistent data but there are a few points that I need to underlie and request revision.

- Panels of Figure 1 and Figure 2 have largely overlapping data for viability, ER stress and apoptosis for L-glu and L-glu SCF. They should be unified.
- Writing receptor tyrosine kinase c-kit (c-KIT) as abbreviation should be mentioned in the first place
- Isolation protocol for hippocampal neurons from mice is not written or cited. This should be included.
- “…c-Kit inhibitor ISC CHOP and…” should be fixed.
- Image quality is so bad for microscope images, that I can not review them. This can be not related to original images maybe due to compression while making the pdf.
- Figure 1legend 1A. “Cell viability was measured using an MTT assay after treatment with 20 μM ginsenoside” ginsenoside? There is something wrong here, that should be fixed.
- The treatment section shows as 24h treatment used as endpoint but Figure A shows up to 72h. Thus I cannot understand the treatment time for the BrdU assay. It should be written clearly. MTT and BrdU methods should be revised.
- How the apoptosis assay was evaluated is not clear. Annexin + and PI + cells were analyzed as apoptotic or just Annexin + cells? This should be indicated.
- Kdas are not indicated for protein bands, figures should be revised.
- I am not so sure that using skimmed milk is the correct protocol for visualizing phospo-proteins. It should be checked. If phospo-proteins are shown in the same membrane, the wb protocol section also should have an appropriate stripping protocol.
- I understand making primary neuronal culture is hard but just having gene expression levels and not having any comparable protein expression level results from the primary model to HT22 cells is the most vulnerable side of the study. Thus, this shortcoming in the study should be added to the discussion.
- In the supplementary materials, I can not see any uncropped blot. Maybe this is not needed by the journal. I can not evaluate the correctness of Kdas.
- In page 19 “…β-dependent…” should be “…Aβ-dependent…”
- Page 19 “Our study provides novel evidence for the first time that SCF can improve AD by regulating ER stress-associated apoptosis” This sentence is not supported by the data. I think this sentence should be corrected because it contains an expression outside the purpose and scope of the study. Just showing that the ER stress is reduced in the cell model carrying AD does not mean improving AD.
- Page 20 “Our findings are consistent with previous researches.” Which researches? They are not cited. It should be fixed.
