# Supplementary material for: Stem cell factor restrains endoplasmic reticulum stress-associated apoptosis through c-Kit receptor activation of JAK2/STAT3 axis in hippocampal neuronal cells
Source: PLoS One. 2024 Nov 15;19(11):e0310872. doi: 10.1371/journal.pone.0310872 (PMC11567559; doi:10.1371/journal.pone.0310872)
Supplement: Attachment — Submitted filename: Comments PLOSONE-PONE-D-24-18303.docx [file pone.0310872.s007.docx]

Dear Editor,

The study titled "***Stem cell factor restrains endoplasmic reticulum stress-associated apoptosis through c-kit receptor activation of JAK2/STAT3 axis in hippocampal neuronal cells***" is a well-designed article that clearly explains the purpose and study methods to the reader-scientists. The work can be accepted without requiring revision. However, it is recommended that authors make a few simple suggestions at the proof-reading stage, if appropriate for the Editor.

The author should add introduction part how to manage these inhibitors as AD theraputics and emphasis its importance more.

Please correct in Material and Methods part test names fully for instance “**3-(4,5)-dimethylthiahiazo (-z-y1)-3,5-di- phenytetrazoliumromide (MTT) Test” or “5-Bromo-2'-deoxyuridine (BrdU) Test”**

Figures microscope photos does not look clear, a clearer image can be added for all figures
