# Supplementary material for: Stem cell factor restrains endoplasmic reticulum stress-associated apoptosis through c-Kit receptor activation of JAK2/STAT3 axis in hippocampal neuronal cells
Source: PLoS One. 2024 Nov 15;19(11):e0310872. doi: 10.1371/journal.pone.0310872 (PMC11567559; doi:10.1371/journal.pone.0310872)
Supplement: Attachment — Submitted filename: Response to Reviewers2 Comments PLOSONE-PONE-D-24-18303.docx [file pone.0310872.s008.docx]

We extend our profound appreciation to the editor expert for their meticulous and outstanding efforts. We are dedicated to adhering to the editor's guidance and make improvements to the manuscript to enhance its quality.

The study titled "***Stem cell factor restrains endoplasmic reticulum stress-associated apoptosis through c-kit receptor activation of JAK2/STAT3 axis in hippocampal neuronal cells***" is a well-designed article that clearly explains the purpose and study methods to the reader-scientists. The work can be accepted without requiring revision. However, it is recommended that authors make a few simple suggestions at the proof-reading stage, if appropriate for the Editor.

- The author should add introduction part how to manage these inhibitors as AD theraputics and emphasis its importance more.

Answer: Thanks for your nice suggestions. The corresponding content had been added to the text and its importance had been emphasized in the discussion section.

- Please correct in Material and Methods part test names fully for instance “**3-(4,5)-dimethylthiahiazo (-z-y1)-3,5-di- phenytetrazoliumromide (MTT) Test” or “5-Bromo-2'-deoxyuridine (BrdU) Test”**

Answer: Thanks for your reminder. All test names had been revised to the “full name (abbreviation)” format.

- Figures microscope photos does not look clear, a clearer image can be added for all figures.

Answer: Thanks for your kind reminder. We had adjusted and improved the pixel and sharpness of the microscope images, and uploaded the original data of the microscope images in the supplementary data.
